# Supplementary material for: Convergent Akt activation drives acquired EGFR inhibitor resistance in lung cancer
Source: Nat Commun. 2017 Sep 4;8:410. doi: 10.1038/s41467-017-00450-6 (PMC5583255; doi:10.1038/s41467-017-00450-6)
Supplement: Supplementary file 1 — Supplementary Information [file 41467_2017_450_MOESM1_ESM.pdf]

Description of Supplementary Files

File Name: Supplementary Information
Description: Supplementary Figures and Supplementary Tables

File Name: Peer Review File

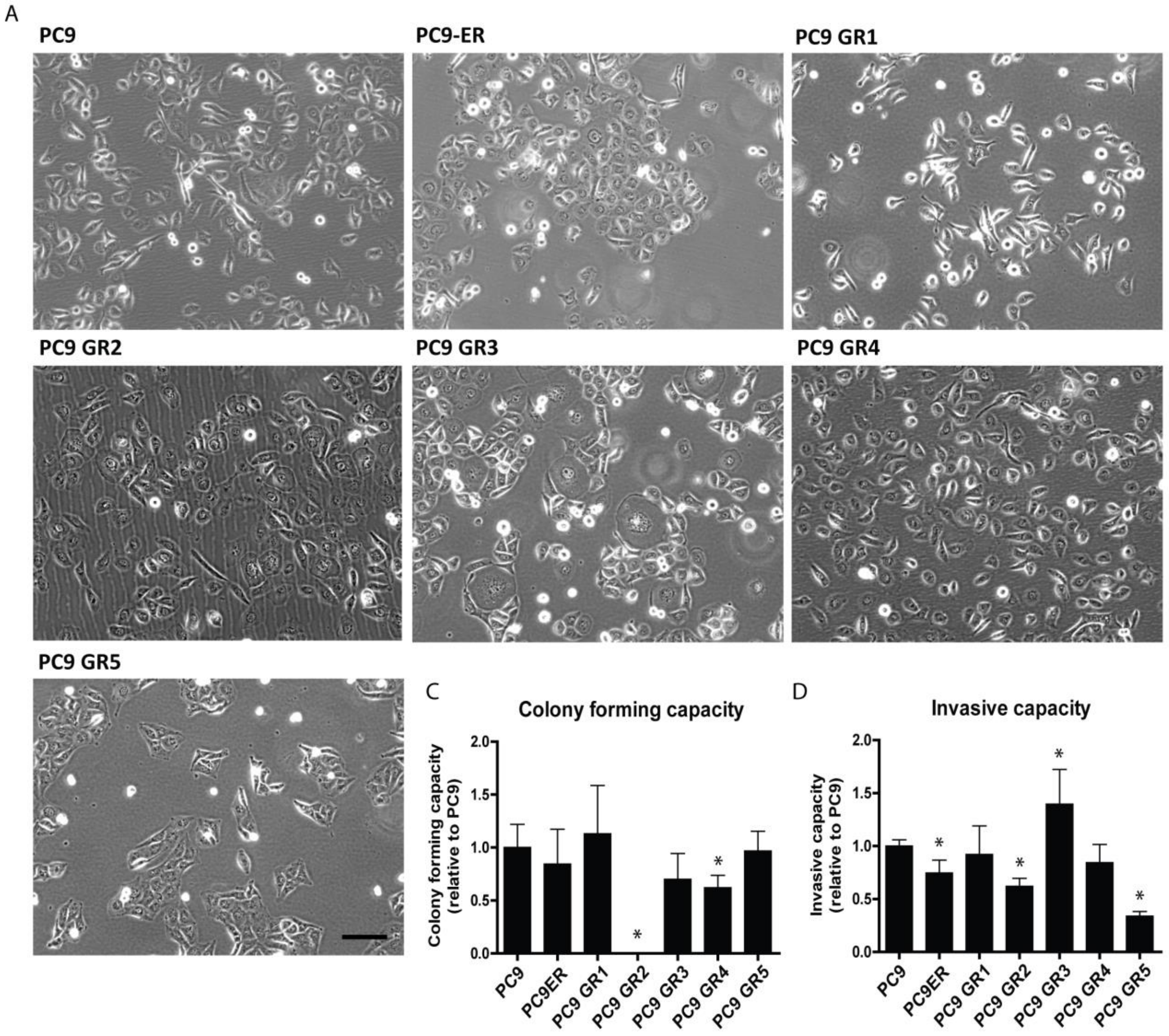

B

Cell line	Doubling time (h)
PC9	21
PC9-ER	23
PC9-GR1	21
PC9-GR2	20
PC9-GR3	23
PC9-GR4	29
PC9-GR5	22

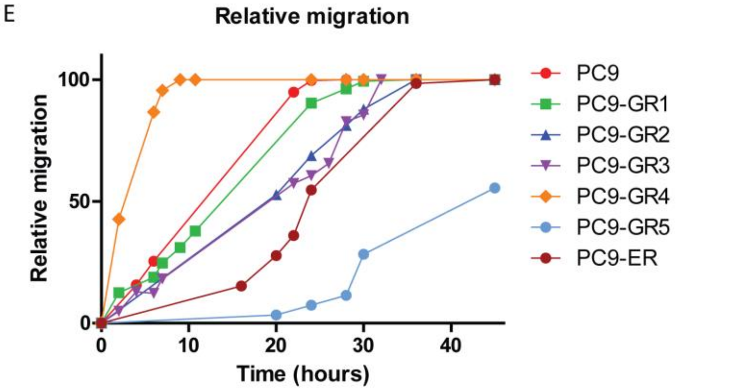

Supplementary Figure 1. Characterization of the EGFR TKI resistant PC9 derived cell lines. (**a**) Microscopic photographs of the parental and resistant PC9 cells. Scale bar, 100 µm. (**b**) Doubling times. (**c**) Colony formation capacity in soft agar of the EGFR TKI resistant cell lines compared to parental PC9. The asterisks indicate significant differences ($p<0.05$ in Student's t test). (**d**) Basement membrane invasive capacity of the EGFR TKI resistant cell lines compared to parental PC9. The asterisks indicate significant differences ($p<0.05$ in Student's t test). (**e**) Migration expressed as % closure of the initial 0.68 mm^2 cell-free region.

a

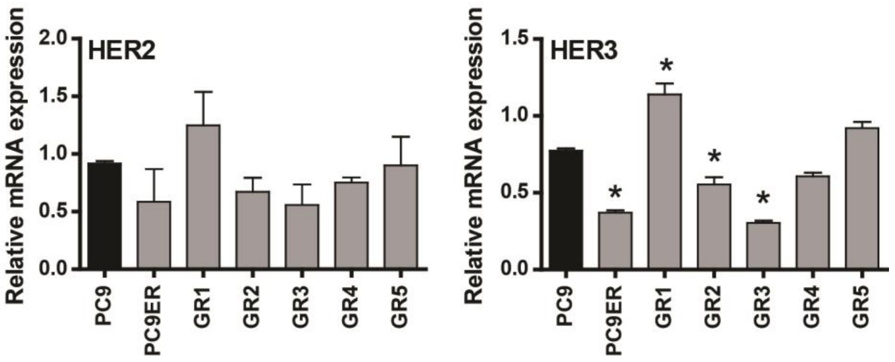

Supplementary Figure 2. RNA expression of HER2 and HER3 in PC9 and its derived resistant cell lines. Data are based on three independent experiments. Asterisks indicate significant difference in ANOVA 1-way test ($p<0.05$) between PC9 and the resistant cell lines.

a

b

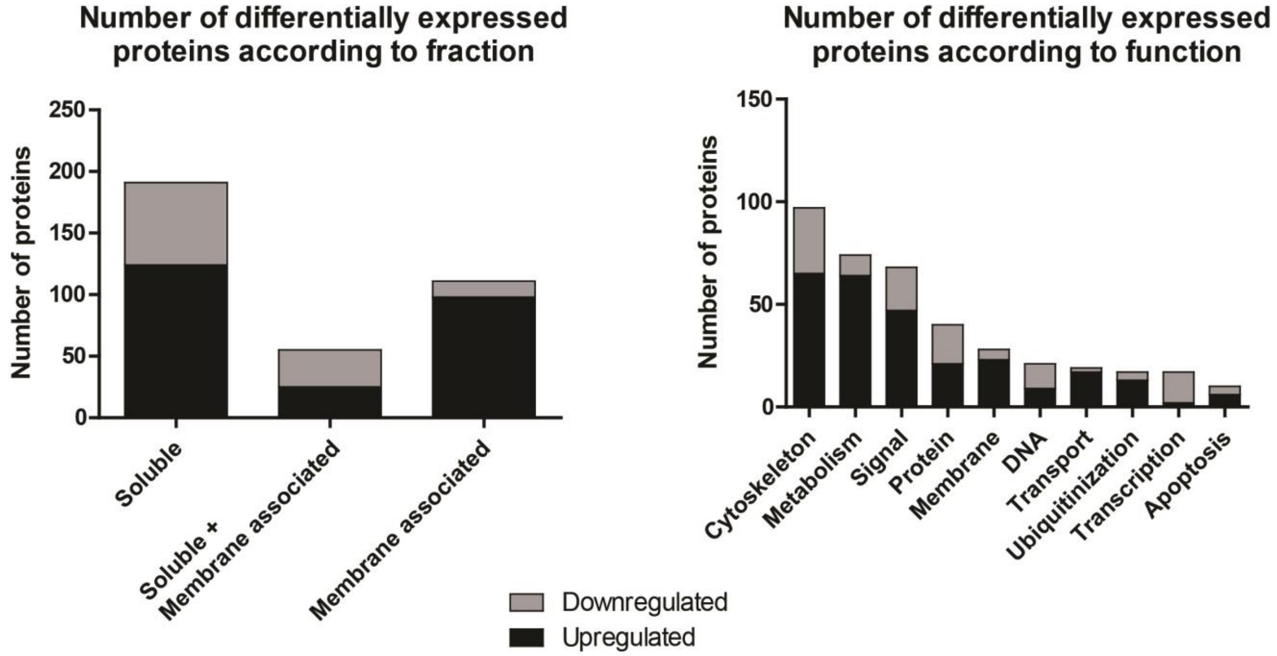

c

Functional category	Also includes proteins involved in
**Cytoskeleton**	Motility, invasiveness, migration, extracellular matrix
**Metabolism**	
**Signal transduction**	
**Protein modification**	Protein synthesis, protein folding, heat shock proteins
**Membrane trafficking**	Endocytosis, phagocytosis, exocytosis
**DNA replication**	DNA repair, mitotic spindle assembly, cell cycle control
**Transport**	Ca^{2+} channels
**Ubiquitinization**	Proteasome
**Transcription**	RNA processing and stabilization
**Apoptosis**	Senescence, stem cells

Supplementary Figure 3. Total number of proteins differentially expressed in the EGFR TKI resistant cell line PC9-ER versus the PC9 parental cells. (**a**) Classified according to fraction. (**b**) Classified according to function. (**c**) Details of the functional classification employed.

a

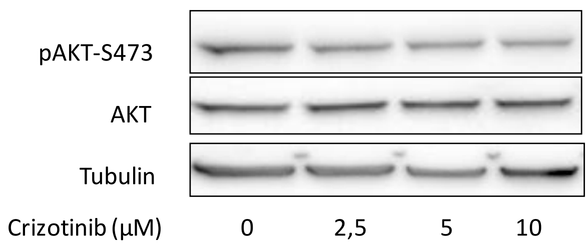

b

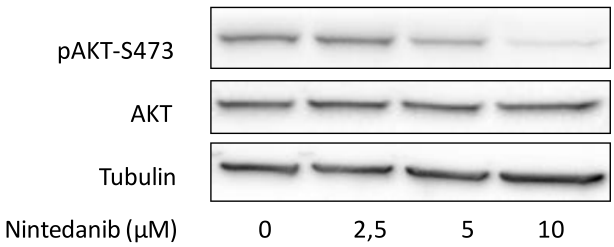

c

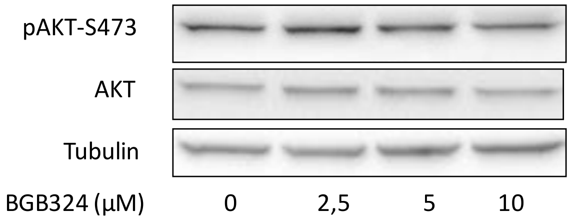

d

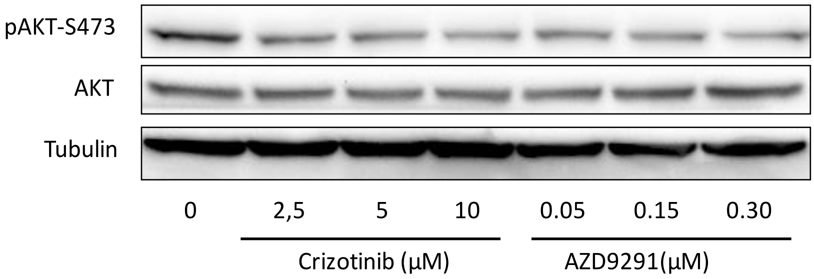

e

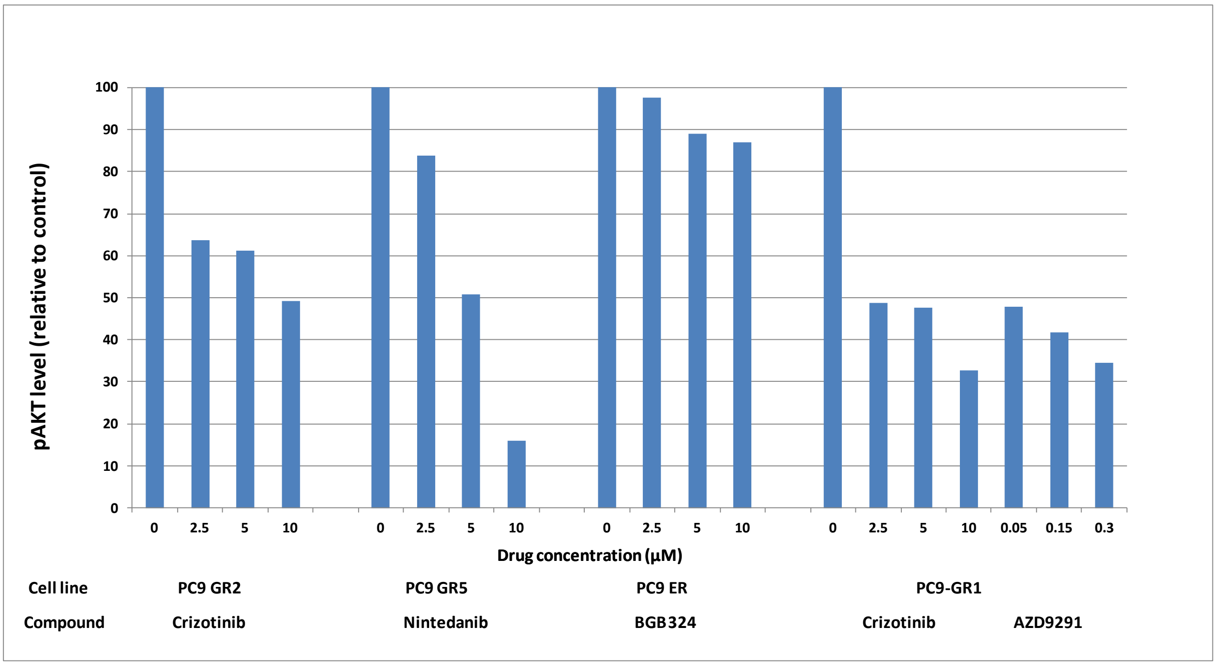

Supplementary Figure 4. Effect of TKIs on Akt phosphorylation in selected resistant PC9 cells. (**a**) Western blot analysis of PC9-GR2 treated for 2 h with crizotinib (2.5-10 µM); (**b**) Western blot analysis of PC9-GR5 treated for 2 h with nintedanib (2.5-10 µM); (**c**) Western blot analysis of PC9-ER treated for 2 h with BGB324 (2.5-10 µM); (**d**) Western blot analysis of PC9-GR1 treated for 2 h with crizotinib or AZD9291 (**e**) quantification of the pAKT bands, normalized to total AKT.

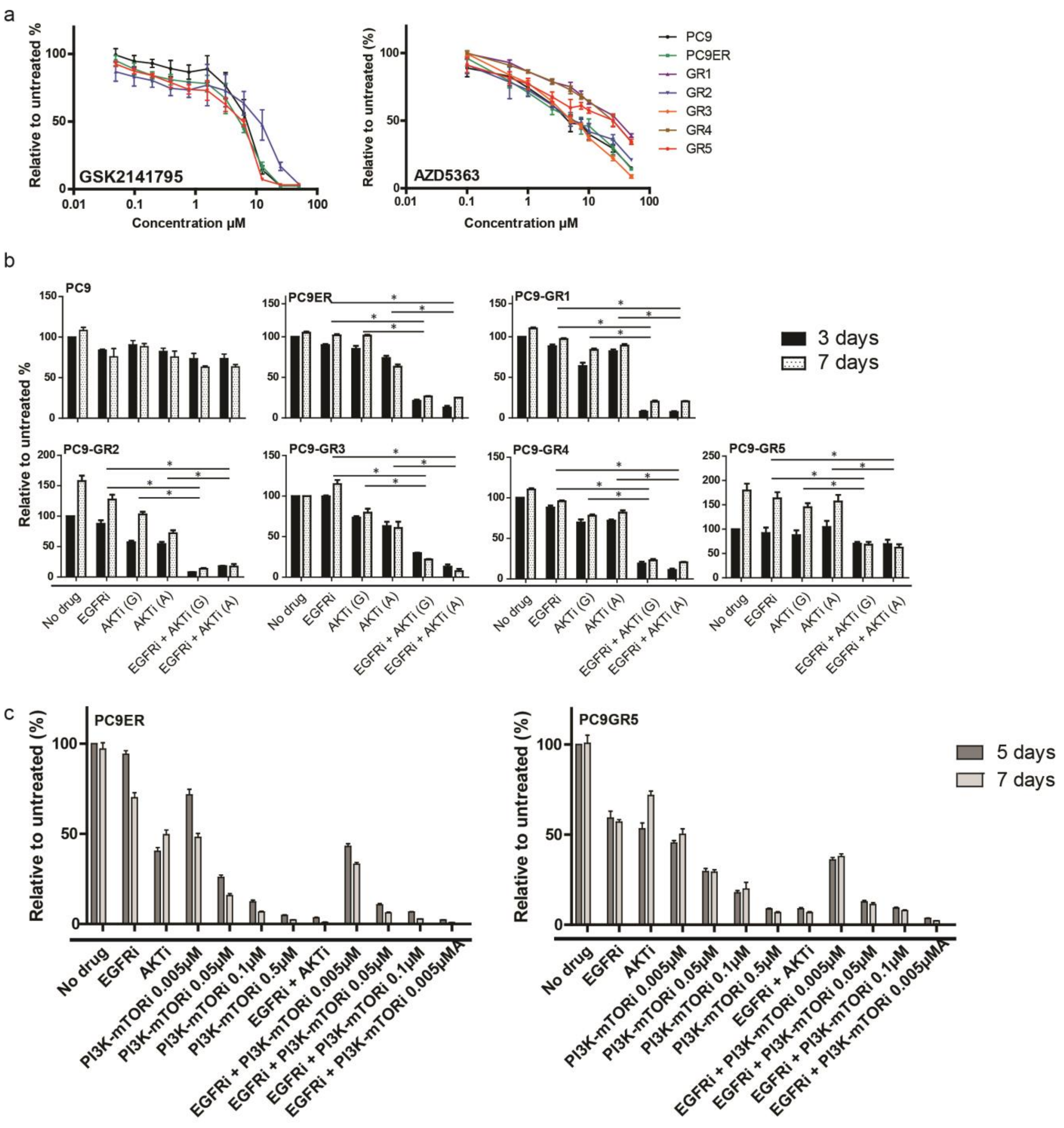

Supplementary Figure 5. Synergistic effects of EGFR TKIs combined with Akt or mTOR inhibitors in EGFR TKI-resistant PC9 cells. (**a**) Growth inhibitory effect of two selective ATP competitive Akt inhibitors, GSK2141795 and AZD5363 were assessed using CellTiterBlue assay. Cells were incubated for 72h. Concentrations of drugs were chosen to be sub-inhibitory to explore the synergistic potential: erlotinib 30 µM; gefitinib 5 µM (except for PC9 where 40nM was used); GSK2141795 (G) 2.5 µM; AZD5363 (A) 35 µM, (except for GR2 and GR5 where 1.25 and 3 µM were employed). Results are mean of seven replicates ±SD. (**b**) CellTiterBlue assay assessing the combinatorial effect of EGFR TKIs combined with Akt inhibitors. "EGFRi" denotes either erlotinib or gefitinib. "AKTi (G)" denotes GSK2141795. "AKTi (A)" denotes AZD5363. Asterisks indicate significant difference in ANOVA 1-way test ($p<0.05$) between the drug combination treated cells and cells treated with either drug alone. For PC9-GR5, significance is only associated with measurements at the day 7 time point. (**c**) CellTiterGlo assay assessing the combinatorial effect of EGFR TKIs combined with the Akt inhibitor GSK2141795 or the PI3K-mTOR inhibitor GSK2126458.

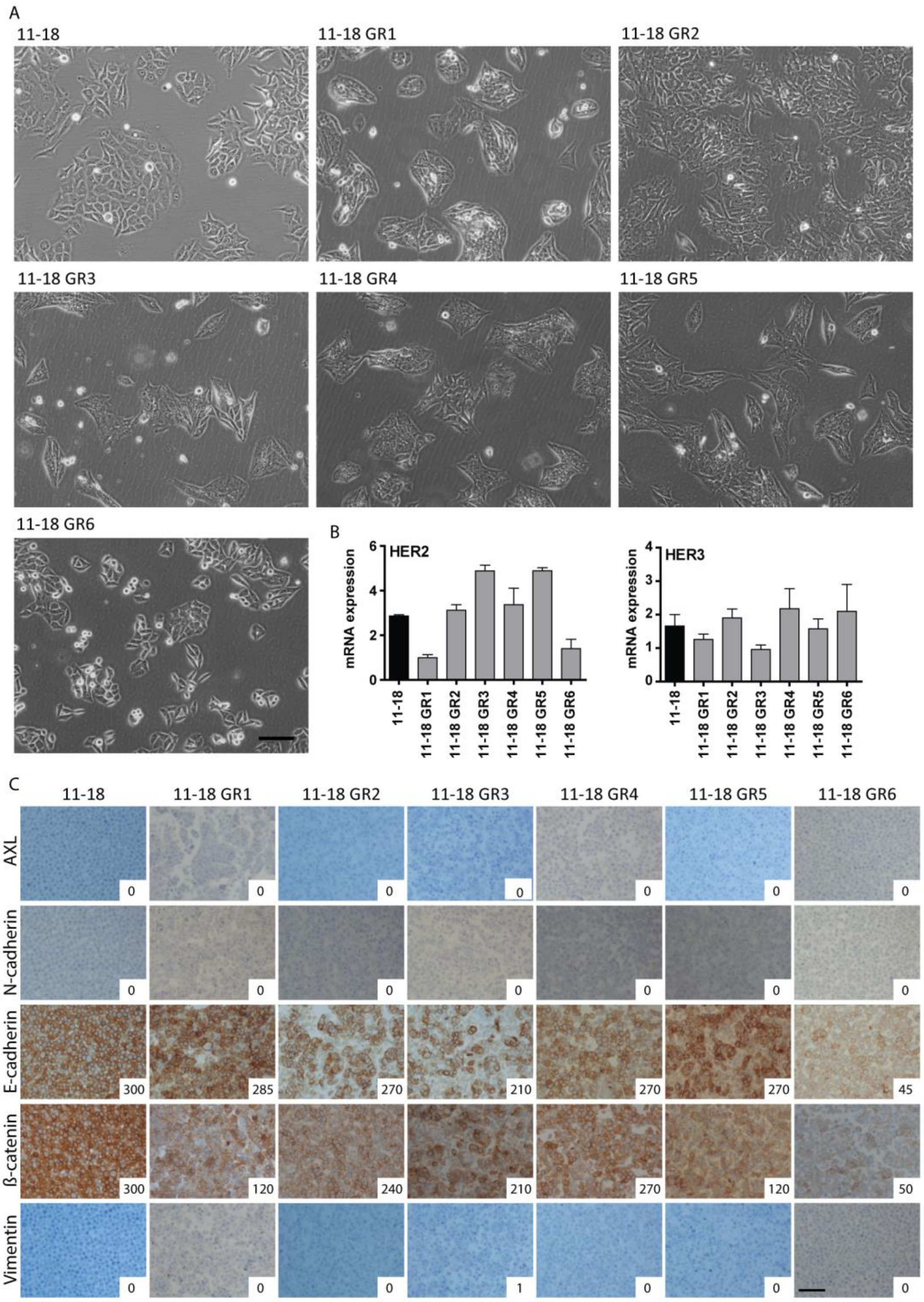

Supplementary Figure 6. Characterization of the EGFR TKI resistant 11-18 derived cell lines. (**a**) Microscopic photographs of the parental and EGFR TKI resistant 11-18 cell lines. Scale bar, 100 µm. (**b**) RNA expression of HER2 and HER3 in 11-18 and its derived resistant cell lines. Data are based on three independent experiments. (**c**) Immunohistochemistry of selected EMT markers in the 11-18 cell lines. 11-18 GR1-6 cell lines exhibit slightly lower E-cadherin and β-catenin expression compared to 11-18. None of the cell lines show expression of AXL, N-cadherin or vimentin. Scale bar, 100 µm.

a

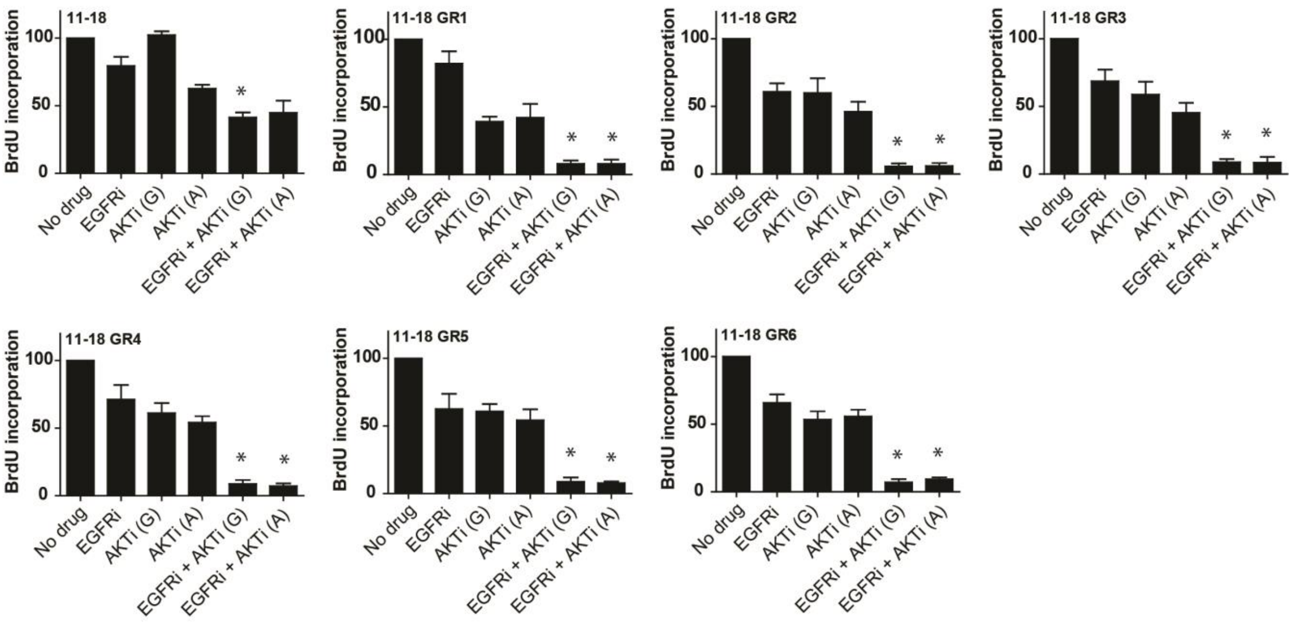

Supplementary Figure 7. Growth inhibitory effect of the combination of gefitinib with either of the two Akt inhibitors, GSK2141795 and AZD5363, on different 11-18 resistant cell lines as evaluated using a BrdU incorporation assays. (**a**) BrdU incorporation assay performed after 96h incubation. Data are mean of triplicates ± SD. Asterisks indicate significant difference in ANOVA 1-way test ($p<0.05$) for the drug combination treated cells compared to cells treated with either drug alone.

a

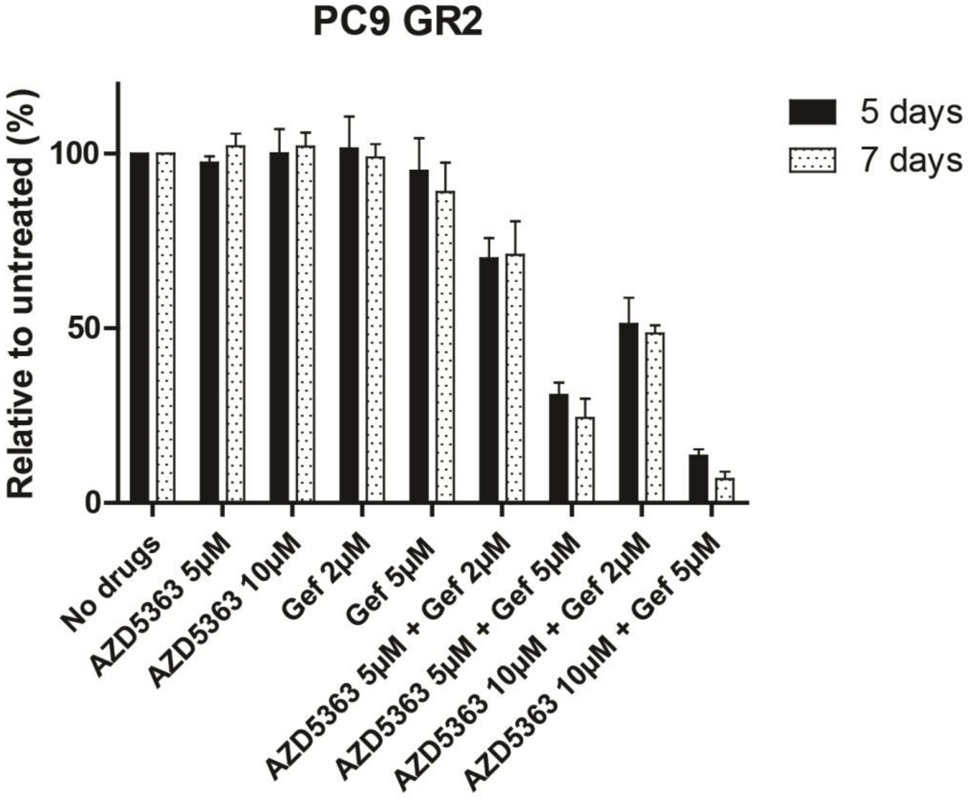

b

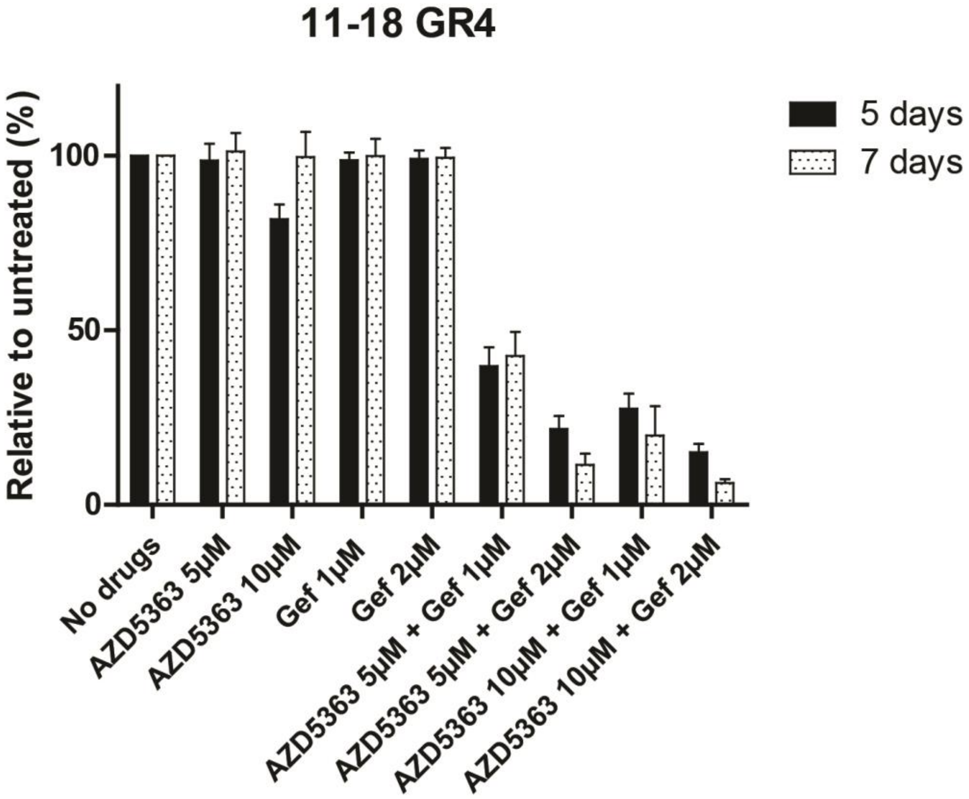

Supplementary Figure 8. Synergistic effects of EGFR TKIs and Akt inhibitors at physiologic concentrations. The combined effects of EGFR inhibitors (gefitinib) and Akt inhibitor (AZD5363) were assessed in the gefitinib-resistant NSCLC cell lines (**a**) PC9 GR2 and (**b**) 11-18 GR4 by crystal violet viability assay performed over seven days.

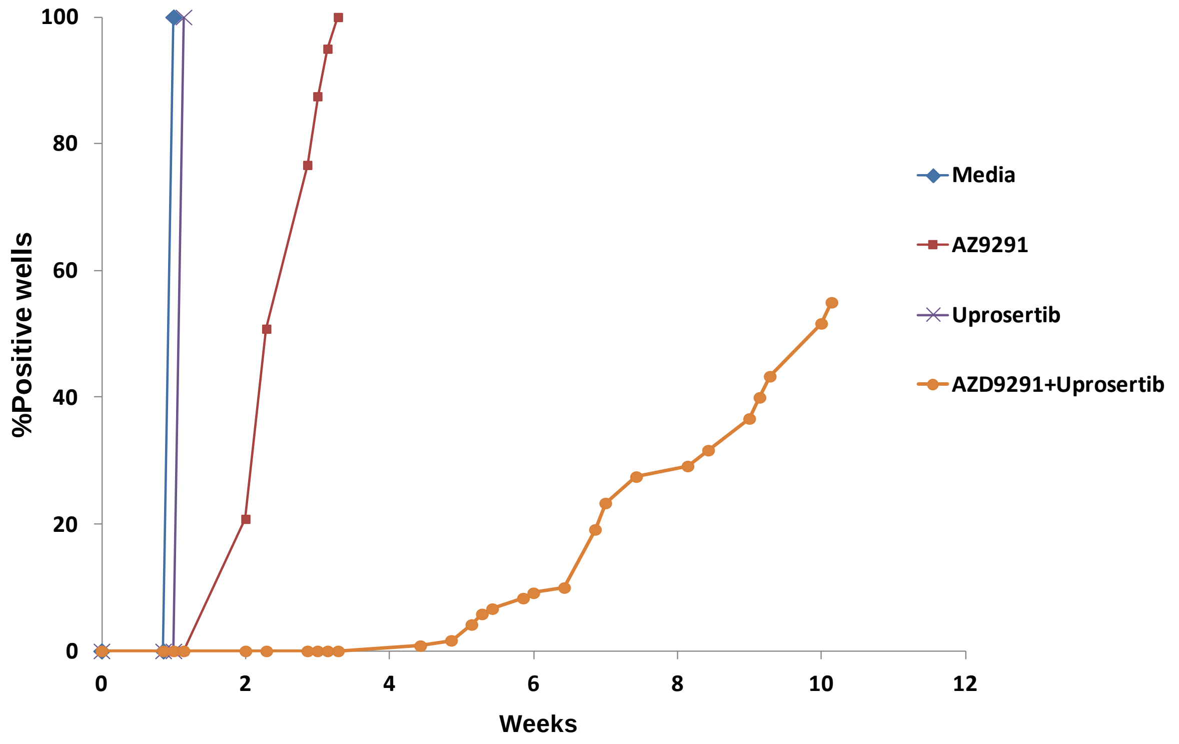

Supplementary Figure 9. **Effects of Akt inhibitor (uprosertib) on the acquisition of resistance to osimertinib in PC9-GR4 cells.** Cells were treated with DMSO control (media), 20 nM osimertinib (AZD9291), 250 nM uprosertib (GSK2141795) or both (2 plates = 120 wells per treatment). The percentage of wells at 50% or greater confluence (positive wells) was assessed daily. The y-axes indicate weeks to confluence.

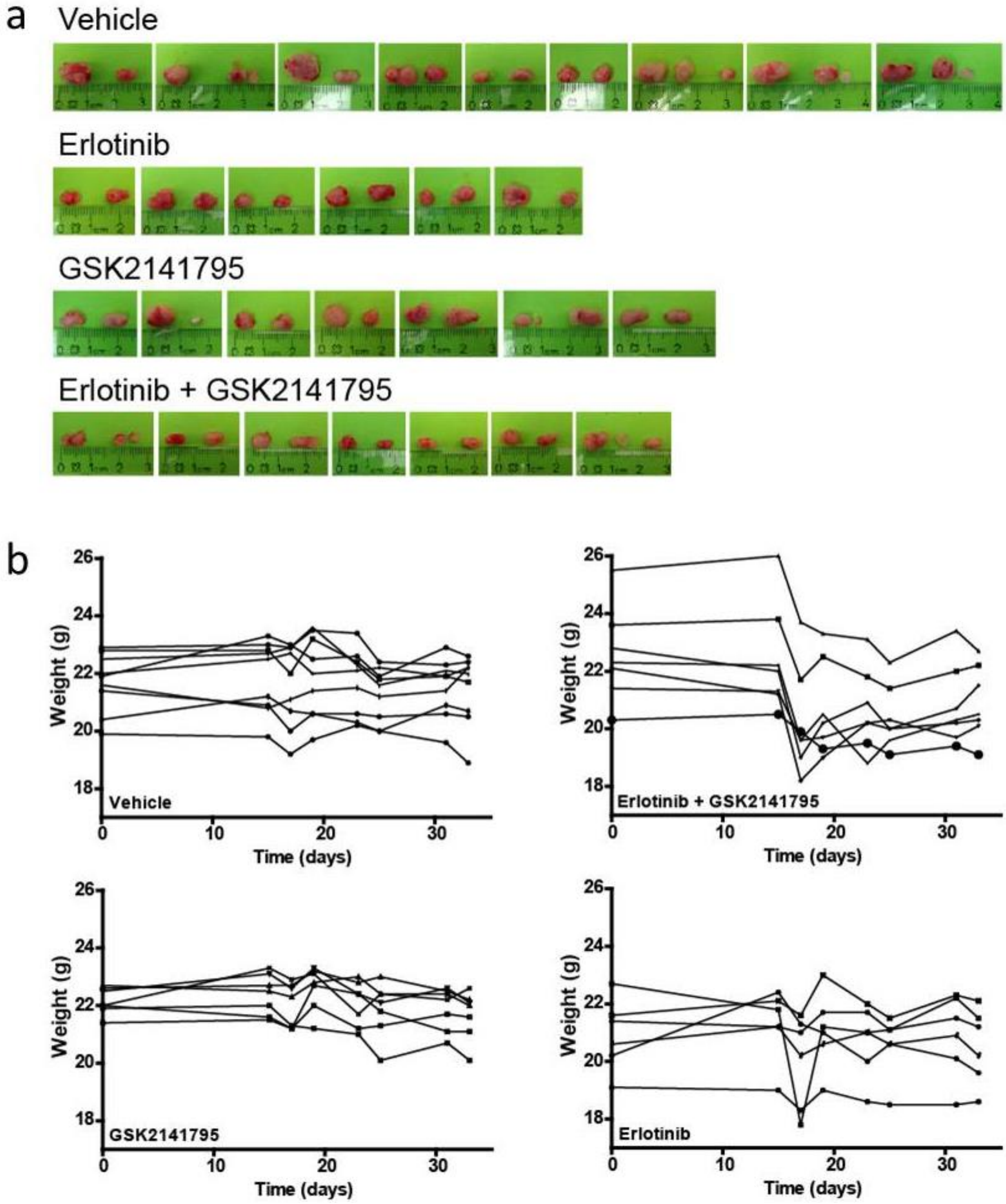

Supplementary Figure 10. Assessment of the *in vivo* effect of erlotinib combined with GSK2141795. (**a**) Photographs of tumors after being excised from mice. Each mouse harbored two tumors. (**b**) Assessment of the bodyweight of the animals during the tumor cell transplantation and treatment period.

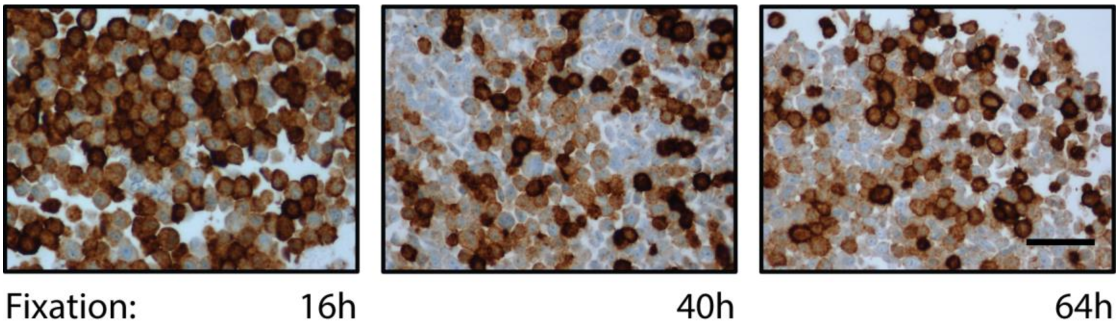

Supplementary Figure 11. Stability of pAkt in immunohistochemical analysis. PC3 cancer cells were fixed with formaldehyde 4x at different time-points (16h, 40h and 64h) to demonstrate that phospho-AKT staining is robust. Phospho-AKT is observed in the cytoplasm of PC3 cell line and with high intensity staining independently of the fixation time. For each experiment, a sample with either an isotype-matched antibody or no primary antibody was included as control. For validation of the pAKT antibody staining a panel of breast cancers with known pAKT levels was used. Scale bar, 100 µm. Magnification 100x.

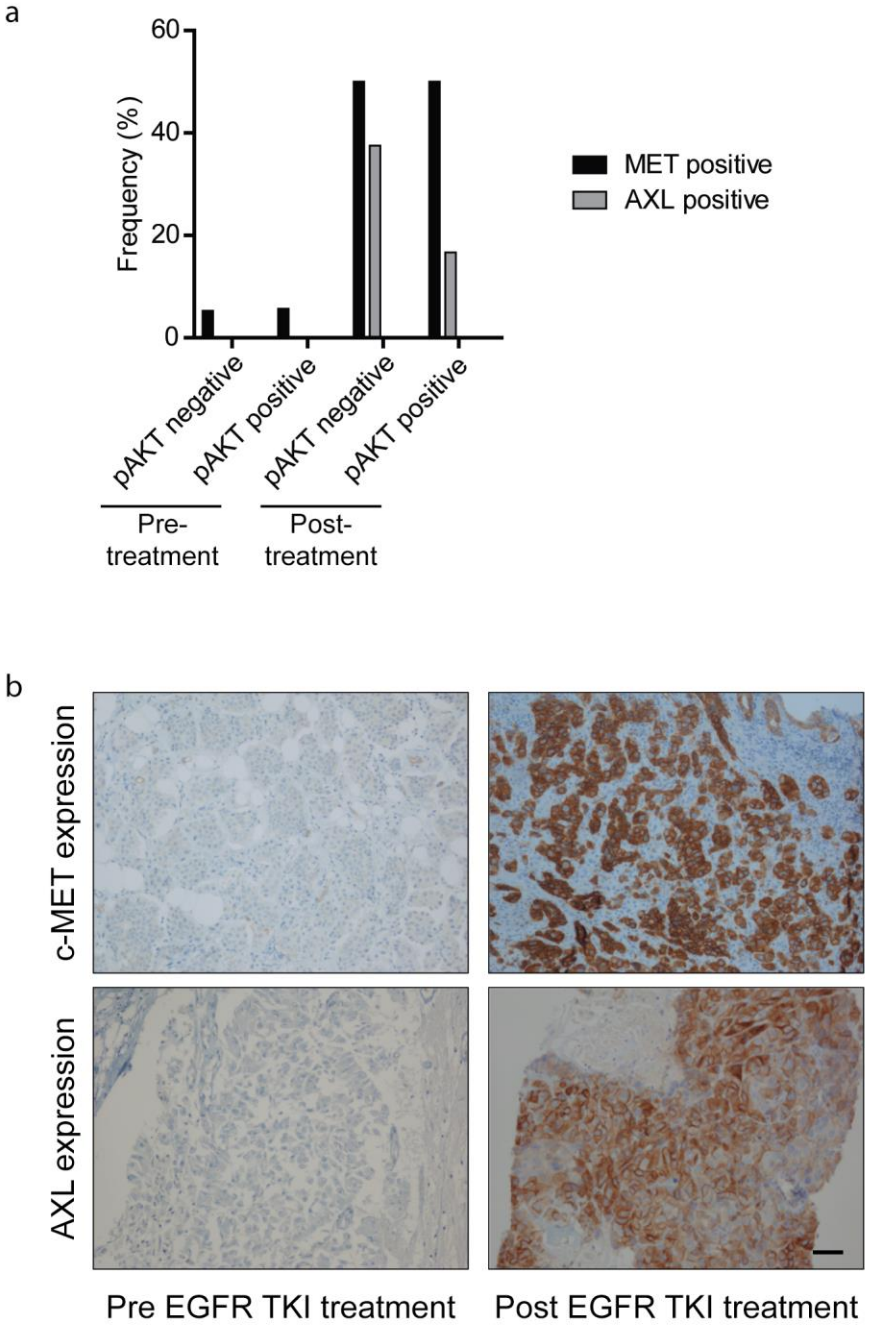

Supplementary Figure 12. Assessment of AXL and c-MET expression in pre- and post-treatment clinical samples. (**a**) Bar graph showing percentage of c-MET-positive and AXL-positive within the pAKT-positive or -negative EGFR-mutant NSCLC patients at baseline and after progressing on treated with first line EGFR-TKIs. (**b**) Representative negative and positive immunohistochemical staining of c-MET and AXL. Scale bar, 100 µm.

Supplementary Table 1. Genotyping of parental PC9 and derived cell lines.

Gene	Codons/Exons analyzed	PC9	PC9-ER	PC9-GR1	PC9-GR2	PC9-GR3	PC9-GR4	PC9-GR5
EGFR*	Exon 19	Del 15 pb (p.E746-A750)	Del 15 pb (p.E746-A750)	Del 15 pb (p.E746-A750)	Del 15 pb (p.E746-A750)	Del 15 pb (p.E746-A750)	Del 15 pb (p.E746-A750)	Del 15 pb (p.E746-A750)
	Exon 20	wt	wt	T790M	wt	wt	T790M	wt
	Exons 18, 21	wt	wt	wt	wt	wt	wt	wt
TP53	Exon 7	p.R248Q	p.R248Q	p.R248Q	p.R248Q	p.R248Q	p.R248Q	p.R248Q
KRAS	Exon 2	wt	wt	wt	wt	wt	wt	wt
BRAF	Codon 600	wt	wt	wt	wt	wt	wt	wt
CTBNN1	Exon 3	wt	wt	wt	wt	wt	wt	wt
PIK3CA	Codons 542, 545, 1047	wt	wt	wt	wt	wt	wt	wt

***** The resistant cell lines were characterized for hotspot mutations in several genes (EGFR, KRAS, BRAF, PIK3CA, TP53, CTNNB1) (Supplementary table 1) by standard PCR followed by Sanger sequencing. To further authenticate cell line identity, short tandem repeat (STR) analysis was performed using the Cell ID™ System (G9500, Promega, Madison, USA) as described by the manufacturer. Ten specific loci of the human genome are PCR amplified and analyzed by capillary electrophoresis. All resistant clones of this study to have the same allelic sizes at all ten loci as the parental cells.

Supplementary Table 2. Sensitivity of parental PC9 and derived cell lines to EGFR TKI.

	Gefitinib		Erlotinib		Afatinib		Dacomitinib		AZD9291	
**Cell line**	IC50 (µM)	% (1 µM)	IC50 (µM)	% (1 µM)	IC50 (µM)	% (1 µM)	IC50 (µM)	% (1 µM)	IC50 (µM)	% (1 µM)
**PC9**	0.04	2%	0.0049	2%	0.003	17%	0.007	24%	0.1	20%
**PC9ER**	12.6	88%	28.6	95%	3.4	83%	2.8	98%	3.1	100%
**PC9-GR1**	12.2	96%	4.83	98%	0.3	25%	0.3	27%	0.1	22%
**PC9-GR2**	14.9	85%	25.0	87%	3.9	84%	2.9	73%	2.4	83%
**PC9-GR3**	15.3	83%	33.7	100%	5.8	100%	3.5	89%	4.6	100%
**PC9-GR4**	6.5	91%	3.8	92%	0.5	30%	0.3	28%	0.2	23%
**PC9-GR5**	18.1	86%	21.8	96%	2.0	58%	2.1	64%	4.2	75%

% (1 µM) indicates the percentage of surviving cells at 1 µM of drug. Values are the mean of a minimum of three replicate experiments.

Supplementary Table 3. Summary of mechanisms of resistance in PC9-derived EGFR TKI-resistant cell lines. T790M mutation was assessed by sequencing. AXL overexpression was assessed by IHC, qRT-PCR and Western blotting. FGFR1 overespression was assessed by qRT-PCR and Western blotting. MET activation was assessed by qRT-PCR and Western blotting. EphA2 activation was assessed by Western blotting.

Cell line	T790M mutation	AXL overexpression	FGFR1 overexpression	MET activation	EphA2 activation
**PC9-ER**	-	+	-	-	+
**PC9-GR1**	+	-	-	+	+
**PC9-GR2**	-	+	-	+	-
**PC9-GR3**	-	+	-	-	-
**PC9-GR4**	+	+	-	-	+
**PC9-GR5**	-	+	+	-	-

Supplementary Table 4. Synergism between EGFR TKIs and Akt inhibitors. Effect of the drug combination was determined to be additive (A) or synergistic (S) for the following assays: crystal violet, CellTiterBlue and BrdU incorporation. Values of ≥1 are additive, whereas values <1 are synergistic.

	PC9	PC9-ER	PC9-GR1	PC9-GR2	PC9-GR3	PC9-GR4	PC9-GR5
**Synergistic coefficient Crystal violet**	1.012	0.000	0.079	0.189	0.196	0.152	0.801
**Result**	A	S	S	S	S	S	S
**Synergistic coefficient CellTiterBlue**	1.020	0.269	0.269	0.164	0.237	0.338	0.516
**Result**	A	S	S	S	S	S	S
**Synergistic coefficient BrdU incorporation**	0.251	0.058	0.052	0.165	0.358	0.100	0.022
**Result**	S	S	S	S	S	S	S

	11-18	11-18 GR1	11-18 GR2	11-18 GR3	11-18 GR4	11-18 GR5	11-18 GR6
**Synergistic coefficient CellTiterBlue**	0.68	0.12	0.13	0.11	0.14	0.08	0.10
**Result**	S	S	S	S	S	S	S
**Synergistic coefficient BrdU incorporation**	0.901	0.229	0.211	0.271	0.186	0.229	0.255
**Result**	A	S	S	S	S	S	S

Supplementary Table 5. Synergism for EGFR inhibitors combined with either GSK2141795 or GSK2126458. Effect of the drug combination was determined to be additive (A) or synergistic (S). Values of ≥1 are additive, whereas values <1 are synergistic.

		5 days		7 days	
		**PC9ER**	**PC9GR5**	**PC9**	**PC9GR5**
**Erlotinib + GSK2141795 (2.5μM)**	**Synergistic coefficient**	0.094	0.283	0.027	0.167
	**Result**	S	S	S	S
**Erlotinib + GSK2126458 (0.005μM)**	**Synergistic coefficient**	0.638	1.342	0.956	1.334
	**Result**	S	A	D	A
**Erlotinib + GSK2126458 (0.05μM)**	**Synergistic coefficient**	0.442	0.725	0.54	0.682
	**Result**	S	S	S	S
**Erlotinib + GSK2126458 (0.1μM)**	**Synergistic coefficient**	0.578	0.884	0.582	0.706
	**Result**	S	S	S	S
**Erlotinib + GSK2126458 (0.5μM)**	**Synergistic coefficient**	0.508	0.695	0.479	0.579
	**Result**	S	S	S	S

Supplementary Table 6. IC50 for gefitinib for the 11-18 parental and resistant cell lines.

Cell line	IC50 Gefitinib (μM)
**11-18**	0.3
**11-18 GR1**	27.1
**11-18 GR2**	8.0
**11-18 GR3**	27.4
**11-18 GR4**	20.6
**11-18 GR5**	20.8
**11-18 GR6**	8.1

Supplementary Table 7. Patient characteristics of 75 patients with EGFR mutant NSCLC treated with first line EGFR TKIs (pre-treatment samples).

**Clinical characteristics of the 75 patients (pre-treatment samples)**	
**Sex—no. (%)**	
Male	21 (28.0)
Female	54 (72.0)
**Age—yr**	
Median	64
Range	35−89
**ECOG*performance status — no. (%)**	
0	18 (24.0)
1	37 (49.3)
2	17 (22.7)
3	3 (4.0)
**Smoking status — no. (%)**	
Never smoked	50 (66.7)
Former smoker	20 (26.7)
Current smoker	5 (6.7)
**Disease stage — no. (%)**	
IIIB	14 (18.7)
IV	61 (81.3)
**Brain metastasis — no. (%)**	
No	52 (69.3)
Yes	23 (30.7)
**Bone metastasis— no. (%)**	
No	42 (56.0)
Yes	33 (44.0)
**Type of EGFR mutation— no. (%)**	
Exon 19 deletion	51 (68.0)
L858R	23 (30.7)

Other**	1 (1.3)
**Type of EGFR TKI— no. (%)**	
Erlotinib	44 (58.7)
Gefitinib	29 (38.7)
Afatinib	2 (2.6)

* ECOG, Eastern Cooperative Oncology Group

**Other, exon 20 insertion

Supplementary Table 8. Evaluation of common mechanisms of resistance to EGFR TKI in pre-treatment clinical samples. The expression of AXL, MET, HER2 and FGFR1 were evaluated in pre-treatment tumor samples where additional tissue was available and correlated with the pAkt expression. Hotspot mutations in *PIK3CA* were also tested using DNA extracted from Hematoxylin & Eosin or IHC slides.

ID	pAKTlevel pos/neg	MET expression pos/neg	AXL expression pos/neg	FGFR1 expression pos/neg	HER2 expression pos/neg	PIK3CA genotype
1	NEG	NEG	NEG	POS	NEG	ND
2	NEG	NEG	NEG	POS	NEG	wt
3	NEG	NEG	NEG	NEG	NEG	wt
4	NEG	NEG	NEG	POS	NEG	wt
5	NEG	NEG	NEG	NEG	NEG	wt
6	NEG	NEG	NEG	NEG	NEG	E542K
7	NEG	NEG	NEG	POS	NEG	wt
8	NEG	NEG	NEG	NEG	NEG	wt
9	NEG	NEG	NEG	NEG	NEG	wt
10	NEG	NEG	NEG	NEG	NEG	wt
11	NEG	NEG	NEG	POS	NEG	wt
12	NEG	NEG	NEG	NEG	NEG	wt
13	NEG	NEG	NEG	NEG	NEG	wt
14	NEG	NEG	NEG	POS	NEG	wt
15	NEG	NEG	NEG	POS	NEG	wt
16	NEG	NEG	NEG	NEG	NEG	wt
17	NEG	NEG	NEG	POS	NEG	wt
18	NEG	NEG	NEG	NEG	NEG	wt
19	NEG	POS	NEG	NEG	NEG	wt
20	NEG	NEG	NEG	POS	NEG	wt
21	NEG	NEG	NEG	NEG	NEG	wt
22	NEG	ND	NEG	ND	ND	wt
23	NEG	NEG	NEG	POS	NEG	wt
24	NEG	POS	NEG	ND	ND	ND
25	NEG	NEG	NEG	NEG	NEG	ND
26	NEG	NEG	NEG	NEG	NEG	wt
27	NEG	NEG	NEG	NEG	NEG	wt
28	NEG	NEG	NEG	NEG	NEG	wt
29	NEG	NEG	ND	ND	ND	ND
30	NEG	NEG	ND	ND	ND	wt
31	POS	NEG	NEG	NEG	NEG	ND
32	POS	NEG	NEG	POS	NEG	wt
33	POS	NEG	ND	ND	ND	wt
34	POS	NEG	ND	ND	ND	wt
35	POS	NEG	NEG	POS	ND	wt
36	POS	NEG	NEG	NEG	NEG	E545K
37	POS	POS	ND	ND	ND	wt
38	POS	NEG	NEG	POS	NEG	wt
39	NEG	ND	ND	ND	ND	wt
40	NEG	ND	ND	ND	ND	wt
41	NEG	ND	ND	ND	ND	wt
42	NEG	ND	ND	ND	ND	wt
43	NEG	ND	ND	ND	ND	wt

44	NEG	ND	ND	ND	ND	wt
45	NEG	ND	ND	ND	ND	wt
46	NEG	ND	ND	ND	ND	wt
47	NEG	ND	ND	ND	ND	wt
48	NEG	ND	ND	ND	ND	wt
49	NEG	ND	ND	ND	ND	wt
50	NEG	ND	ND	ND	ND	wt
51	NEG	ND	ND	ND	ND	L1047R
52	NEG	ND	ND	ND	ND	wt
53	NEG	ND	ND	ND	ND	wt
54	NEG	ND	ND	ND	ND	wt
55	NEG	ND	ND	ND	ND	wt
56	NEG	ND	ND	ND	ND	wt
57	NEG	ND	ND	ND	ND	wt
58	NEG	ND	ND	ND	ND	wt
59	NEG	ND	ND	ND	ND	wt
60	NEG	ND	ND	ND	ND	wt
61	NEG	ND	ND	ND	ND	wt
62	NEG	ND	ND	ND	ND	wt
63	NEG	ND	ND	ND	ND	wt
64	NEG	ND	ND	ND	ND	wt
65	NEG	ND	ND	ND	ND	wt
66	NEG	ND	ND	ND	ND	wt
67	NEG	ND	ND	ND	ND	wt
68	NEG	ND	ND	ND	ND	wt
69	NEG	ND	ND	ND	ND	wt
70	NEG	ND	ND	ND	ND	wt
71	NEG	ND	ND	ND	ND	wt
72	NEG	ND	ND	ND	ND	ND
73	NEG	ND	ND	ND	ND	ND
74	NEG	ND	ND	ND	ND	ND
75	NEG	ND	ND	ND	ND	ND

ND, not determined.

Supplementary Table 9. pAKT level and T790M status of EGFR TKI post-treated samples.

Patient #	pAKT pos/neg	pAKT histoscore	T790M
1	POS	210	POS
2	POS	120	POS
3	NEG	0	POS
4	NEG	0	NEG
5	POS	300	POS
6	POS	285	POS
7	NEG	45	POS
8	POS	240	POS
9	POS	180	POS
10	NEG	0	NEG
11	NEG	0	POS
12	POS	75	NEG
13	POS	105	POS
14	POS	65	NEG
15	NEG	45	NEG

*The histoscore value of 62 was used to classify samples as positive (above 62) or negative (below 62) for pAKT

Supplementary Table 10. Evaluation of common mechanisms of resistance to EGFR TKI in post-treatment clinical samples.

ID	pAKTlevel pos/neg	PIK3CA mutation	T790M mutation	AXL expression pos/neg	MET amplification FISH pos/neg	MET expression pos/neg	Vimentinexpression pos/neg	E-cad expression pos/neg
1	POS	wt	+	POS	NEG	NEG	NEG	POS
2	POS	wt	+	NEG	NEG	NEG	POS	POS
3	NEG	wt	+	NEG	NEG	NEG	POS	POS
4	NEG	ND	-	NEG	ND	POS	POS	POS
5	POS	wt	+	POS	ND	ND	ND	ND
6	POS	wt	+	POS	POS	NEG	POS	POS
7	NEG	wt	+	ND	NEG	NEG	POS	POS
8	POS	wt	+	NEG	POS	NEG	POS	POS
9	POS	wt	+	NEG	POS	POS	NEG	POS
10	NEG	ND	-	NEG	NEG	POS	NEG	POS
11	NEG	L1047R	-	NEG	ND	NEG	NEG	POS
12	POS	wt	-	NEG	POS	POS	ND	ND
13	POS	ND	+	ND	ND	ND	ND	ND
14	POS	ND	-	NEG	NEG	NEG	ND	ND
15	NEG	ND	-	POS	POS	POS	ND	ND

ND, not determined.
